# Small Dense Low-Density Lipoprotein as Biomarker for Atherosclerotic Diseases

**DOI:** 10.1155/2017/1273042

**Published:** 2017-05-07

**Authors:** Ekaterina A. Ivanova, Veronika A. Myasoedova, Alexandra A. Melnichenko, Andrey V. Grechko, Alexander N. Orekhov

**Affiliations:** ^1^Department Development and Regeneration, KU Leuven, 3000 Leuven, Belgium; ^2^Laboratory of Angiopathology, Institute of General Pathology and Pathophysiology, Moscow 125315, Russia; ^3^Centro Cardiologico Monzino IRCCS, 20138 Milan, Italy; ^4^Federal Scientific Clinical Center for Resuscitation and Rehabilitation, 14-3 Solyanka Street, Moscow 109240, Russia; ^5^Institute for Atherosclerosis Research, Skolkovo Innovation Center, Moscow 121609, Russia

## Abstract

Low-density lipoprotein (LDL) plays a key role in the development and progression of atherosclerosis and cardiovascular disease. LDL consists of several subclasses of particles with different sizes and densities, including large buoyant (lb) and intermediate and small dense (sd) LDLs. It has been well documented that sdLDL has a greater atherogenic potential than that of other LDL subfractions and that sdLDL cholesterol (sdLDL-C) proportion is a better marker for prediction of cardiovascular disease than that of total LDL-C. Circulating sdLDL readily undergoes multiple atherogenic modifications in blood plasma, such as desialylation, glycation, and oxidation, that further increase its atherogenicity. Modified sdLDL is a potent inductor of inflammatory processes associated with cardiovascular disease. Several laboratory methods have been developed for separation of LDL subclasses, and the results obtained by different methods can not be directly compared in most cases. Recently, the development of homogeneous assays facilitated the LDL subfraction analysis making possible large clinical studies evaluating the significance of sdLDL in the development of cardiovascular disease. Further studies are needed to establish guidelines for sdLDL evaluation and correction in clinical practice.

## 1. Introduction

High incidence of atherosclerosis and associated cardiovascular diseases (CVD) urges the study of the causes and the risk factors of their development. Atherosclerotic plaque growth is dependent on the uptake of circulating cholesterol by subendothelial cells. Hypercholesterolemia is one of the well-understood risk factors of atherosclerosis, and cholesterol-lowering therapy is widely used in clinical practice for treatment of CVD [1, 2]. However, the CVD risk reduction achieved in most of the clinical studies was not higher than 30% indicative of other important risk factors that have to be taken into account [3–5]. A strong line of evidence demonstrates that the development and progression of atherosclerosis are dependent not only and not so much on the amount as on the specific properties of circulating lipoproteins [6, 7].

Circulating lipoprotein particles vary in size, density, and lipid and apolipoprotein composition and can be separated into several classes based on physical and chemical parameters. Low-density lipoprotein (LDL) is the major source of atherosclerotic lipid storage, whereas high-density lipoprotein (HDL) is not atherogenic, and its level inversely correlates with the atherosclerotic CVD risk [8]. Small dense LDL (sdLDL) is especially common in the serum of atherosclerosis patients and is susceptible to chemical modifications that increase their atherogenicity [9, 10]. The analysis of plasma LDL profile can be performed by ultracentrifugation or gradient gel electrophoresis that can separate the LDL particles based on their density or size correspondingly. Other methods have been used to assess the LDL particle size, charge, or chemical properties and will be discussed later in this review. Currently, the development of cheap and reliable LDL profiling methods for routine clinical practice remains a challenging goal.

Numerous clinical studies have been conducted to establish the link between the composition of circulating LDL particles and the risk of atherosclerosis and CVD development. According to the current consensus, 2 main phenotypes, A and B, are defined based on plasma LDL profile, with intermediate A/B phenotype lying in between [11]. The phenotype A is characterized by the predominance of large buoyant LDL (lbLDL) and the phenotype B by the predominance of sdLDL [12, 13]. Phenotype B was reported in a number of diseases, including metabolic disorders [14, 15], obesity [16, 17], and type 2 diabetes [18, 19] and is considered as a risk factor of coronary heart disease (CHD). Moreover, this phenotype was associated with the elevated plasma triglyceride (TG) level, reduced HDL cholesterol (HDL-C), and high-hepatic lipase activity [20]. The predominance of sdLDL is currently accepted as a risk factor for CVD by the National Cholesterol Education Program (NCEPIII) [21]. Apart from density and size, LDL particles may vary in chemical composition because of a series of modifications that they can undergo in human blood. Among them, lipoprotein(a) (Lp(a)), which contains an additional lipoprotein molecule covalently bound to apolipoprotein B, has been characterized as an additional cardiovascular risk factor [22]. Detection and measurement of modified LDL particles is of special interest, as these types of LDL can be a better marker for increased atherosclerosis, although their content in blood might be scarce in comparison with native LDL.

## 2. LDL Subclasses and Methods of Their Identification

LDL is broadly defined as lipoprotein fraction with density ranging from 1.006 to 1.063 g/ml, which can be isolated by various laboratory methods. This range also includes the intermediate density lipoprotein (IDL) and very low-density lipoprotein (VLDL). More precisely, LDL is known to have a density from 1.019 to 1.063 g/ml. Ultracentrifugation and gradient gel electrophoresis (GGE) with their modifications are widely used for LDL analysis. In most of the studies using these methods, LDL particles are classified into 3 or 4 subclasses, including large (LDL I), intermediate (LDL II), small (LDL III), and, in some studies, very small (LDL IV) LDLs [3, 18]. LDL III and LDL IV (when discerned) are referred to as sdLDL. However, the classification of LDL based on different analytical methods lacks uniformity, and care should be taken while comparing the results of clinical studies employing different methods.

Historically, the first method that allowed separation of different LDL fractions was analytical ultracentrifugation [18, 23–25]. In this method, LDL particles are separated based on their flotation rate (Sf). In studies, where three LDL subclasses are defined, LDL I, II, and III have densities of 1.025–1.034 g/ml, 1.034–1.044 g/ml, and 1.044–1.060 g/ml, respectively [26, 27]. In some studies, very small LDL IV particles are separated. Phenotype pattern A is characterized by the predominance of LDL I and II and atherogenic phenotype pattern B by the predominance (>50%) of LDL III and IV. Different ultracentrifugation methods result in slight variations in the density of the separated LDL. For instance, iodixanol gradient gives lower densities of LDL particles than traditional salt gradient, because the particles maintain their native hydration [3, 28, 29].

Another widely used method of LDL subfraction analysis is GGE under nondenaturating conditions. In this method, LDL subclasses are separated by their electrophoretic mobility, which is determined by the size and shape of the lipoprotein [30]. Studies using GGE separation of LDL define 4 subclasses: LDL I (large LDL, peak diameter 26.0–28.5 nm), LDL II (intermediate LDL, 25.5–26.4 nm), LDL III A and B (small LDL, 24.2–25.5 nm), and LDL IV A and B (very small LDL, 22.0–24.1 nm) [31]. Two phenotypes can be distinguished based on peak LDL particle diameters: >25.5 nm for phenotype pattern A (large and intermediate LDL) and ≤25.5 nm for phenotype pattern B (small and very small LDL). There is a strong correlation between size and density of LDL particles analyzed by ultracentrifugation and GGE, respectively; however, these parameters are not identical. Some authors used tube gel electrophoresis for LDL subfraction analysis for rapid acquisition of quantitative results [32, 33].

Nuclear magnetic resonance (NMR) can be employed for studying of lipoprotein classes in blood plasma, including subclasses of LDL. However, the results of particle size measurement by NMR differ significantly from the GGE data in the same patients and can not be directly compared. sdLDL is determined by NMR as particles with sizes from 18.0 to 20.5 nm [34, 35].

Other methods of LDL fraction analysis include high-performance liquid chromatography (HPLC) with gel filtration columns [36], dynamic light scattering [37, 38], ion mobility analysis [39, 40], and homogenous assay analysis [41]. The latter is of special interest because of its high reproducibility and suitability for large-scale clinical trial use. The homogenous assay for detection of sdLDL-cholesterol was first described by Hirano et al. [41]. Since then, the assay has been modified to simplify the analytical procedure. In the modified method, sdLDL (particle size 15.0–20.0 nm) is separated from lbLDL using detergent and sphingomyelinase treatment, and sdLDL-cholesterol concentration is measured. The method separates sdLDL fraction with a density from 1.044 to 1.063 g/ml using standard clinical laboratory equipment [42, 43]. The comparison of some of the most widely used methods of LDL subclasses analysis is presented in Table 1.

As the clinical and diagnostic significance of LDL subclasses becomes evident, the standardization problem comes into prominence. Different methods of LDL subclass analysis deliver different results, and significant variations are possible even within one method. It is currently difficult to determine which of the existing approaches can be recommended as the most accurate and, at the same time, suitable for clinical use. Moreover, no data is currently available on the comparability of the LDL subfraction analysis methods in terms of predicting CVD outcomes [46]. Therefore, more studies are needed to develop a standard analytical procedure.

## 3. Origins of LDL Subclasses

**Figure 1:**
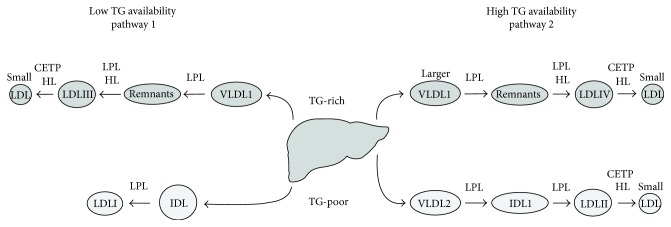
Hypothetical scheme of metabolic origins of LDL subclasses. Two metabolic pathways exist for the production of LDL particles from the precursors secreted by the liver. In case of low TG availability, the liver secretes mostly VLDL1 and IDL as TG-rich and TG-poor lipoprotein particles. These can be modified by LPL and HL to generate LDLI and III particles. In case of high TG availability, a distinct pattern of LDL precursors is secreted, including larger VLDL1 and VLDL2. After these modifications by LPL and HL, they give rise to LDLII and IV particles. After TG transfer to the LDL particles by CETP, they can be further delipidated by HL resulting in the formation of smaller LDL particles. TG: triglycerides; LPL: lipoprotein lipase; HL: hepatic lipase; CETP: cholesterol ester transfer protein.

The exact origins of LDL subclasses remain to be elucidated. Berneis et al. proposed the existence of two pathways dependent on hepatic triglyceride (TG) availability [44]. Two types of precursor lipoproteins (Lp) are secreted from the liver, containing TG-rich or TG-poor apolipoprotein B (apoB). When the TG availability is low, VLDL1 (TG-rich Lp) and IDL2 (TG-poor Lp) are secreted. If the TG availability is high, larger particles are secreted, such as larger VLDL1 (TG-rich Lp) and VLDL2 (TG-poor Lp). TG-poor Lp is a precursor for larger LDL subclasses (LDL I and LDL II), whereas TG-rich Lp is converted into sdLDL subclasses (LDL III and LDL IV) after delipidation by lipoprotein lipase (LPL) and hepatic lipase (HL). Cholesteryl ester transfer protein (CETP) can transfer TG to sdLDL particles that will be further delipidated by HL, resulting in the generation of smaller particles (Figure 1) [3, 44]. This theory advocates the distinct metabolic pathway for sdLDL from liver-secreted precursors and is supported by the results of an interventional human study that demonstrated an inverse correlation between LDL I and LDL III and between LDL II and LDL IV [44, 47]. As a consequence of step-wise modification, sdLDL particles have altered chemical contents, containing decreased amounts of phospholipids (as measured based on apolipoprotein B content), as well as free cholesterol and cholesterol ester, while TG contents remain unaltered [48].

Recent studies suggest that sdLDL can have multiple origins, at least in patients with metabolic disorders. The results of LDL subfraction analysis on days 0 to 7 after apheresis in patients with familial hypercholesterolemia demonstrated that sdLDL rebound dynamics could be best explained by the model, combining the direct pathway and delipidation of lbLDL [49]. The regulation of sdLDL production is likely to be dependent on the current metabolic status. The regulatory role of apoE and apoC-III lipoproteins in the apoB metabolism was studied in a recent work on healthy subjects and patients with hypertriglyceridemia [50]. When plasma TG levels were normal, the liver secreted primarily apoE-containing TG-rich VLDL that was rapidly removed from the circulation. In hypertriglyceridemia, however, the balance was shifted towards apoC-III-containing TG-rich lipoproteins that had longer circulation times and were converted into sdLDL. Clearance of apoE-containing lipoproteins was also reduced. As a result, high rate of sdLDL formation and reduced clearance led to the development of phenotype pattern B with elevated sdLDL levels. These observations highlight the importance of controlling hypertriglyceridemia for reduction of CVD risk. Numerous studies have been conducted to evaluate the effects of lifestyle and dietary changes on TG and sdLDL production and are reviewed elsewhere [51]. Some dietary components, such as omega-3 polyunsaturated fatty acids, were demonstrated to have beneficial effects [51, 52].

LDL particles can be modified by CETP, which is responsible for the exchange of TG and cholesteryl ester between LDL and VLDL and/or HDL and HL. This leads to the production of smaller sdLDL particles. Correspondingly, inhibition of CETP could reduce the sdLDL fraction in individuals with low HDL-C and in healthy premenopausal women [53, 54].

Genetic factors influencing sdLDL production have been studied in recently performed genome-wide association studies (GWAS). It was found that a single nucleotide polymorphism (SNP) in the promoter region of sortilin, a sorting receptor involved in the hepatic release of VLDL, results in alterations in hepatic sortilin synthesis and has an influence on the lipoprotein profile. Very small LDL fraction was increased by 20% in major allele homozygotes as compared to minor allele homozygotes [55]. Other SNPs associated with altered lipoprotein metabolism have been reported in different loci, including CETP, LPL, LIPC, GALNT2, MLXIPL, APOA1/A5, and PCSK7 [40, 56]. Therefore, sdLDL metabolism is dependent on genetic factors that might be considered for the development of novel therapeutic strategies.

## 4. Atherogenic Modifications of sdLDL

The circulation time of sdLDL is longer than that of large LDL particles that are cleared from the bloodstream through the interaction with the LDL receptor [57, 58]. Lipid trapping and accumulation by foam cells in the arterial wall are the key processes that lead to the development and growth of the atherosclerotic plaque. LDL particles are the main source of cholesterol stored in the plaques and their atherogenic properties have been extensively studied. It was demonstrated that native LDL does not cause lipid accumulation in cultured cells, whereas modified particles, such as oxidized, desialylated, glycated, and electronegative LDL, are highly atherogenic [9, 59]. Modified forms of LDL also possess proinflammatory properties and are prone to aggregation and formation of complexes that further increase their atherogenicity.

Oxidation in blood plasma is one of the first atherogenic modifications of LDL particles that have been proposed [9, 60]. Oxidation results in the generation of oxidation-specific epitopes on the LDL particles that induce the immune response and inflammation. Oxidized LDL is recognized by a number of receptors, including CD36 and TLR-4 [61]. Increased susceptibility of sdLDL to oxidation can be explained by its lipid composition [48]. Moreover, sdLDL particles contain less antioxidative vitamins and are therefore more susceptible to oxidation than larger forms of lipoproteins [62].

Enrichment of lipoprotein-associated phospholipase A2 (Lp-PLA2) in LDL particles is known to be associated with cardiovascular disease. High PLA2 contents were described in electronegative LDL and also in advanced atherosclerotic plaques. Inside the lipoprotein particle, this enzyme cleaves oxidized phospholipids, releasing proinflammatory products and further increasing its atherogenicity [63].

Another atherogenic modification of LDL is desialylation, which is performed in blood plasma by trans-sialidase that plays an important role in the metabolism of glycoconjugates [64]. Trans-sialidase transfers the sialic acid moiety from the LDL particle to various acceptors such as plasma proteins, neutral sphingolipids, or gangliosides. It was demonstrated that incubation of purified LDL with blood plasma for several hours leads to a gradual desialylation of the particles [64]. sdLDL have a decreased sialic acid content in comparison to lbLDL in subjects with phenotype pattern B [6, 65]. Desialylation apparently increases the affinity of the sdLDL particles to proteoglycans in the arterial wall. As a result, desialylated sdLDL has a prolonged residence time in the subendothelial space where it can contribute to the lipid storage and atherosclerosis plaque development [66].

ApoB lipoprotein was shown to be preferentially glycated in sdLDL particles as compared to lbLDL both in vitro and in vivo [67, 68], and the level of glycated apoB inversely correlated by particle size measured by NMR [69].

The origins of the elevated electronegative LDL (LDL(−)) levels in the plasma of atherosclerotic patients are not completely understood. Several mechanisms have been proposed, including oxidation, modification of the protein component, and binding to proteoglycans [70]. The relationship of LDL(−) to sdLDL was a subject of several studies. It has been demonstrated that LDL(−) from plasma of healthy individuals was predominant in the dense subfraction, while most of the LDL(−) from patients with hypercholesterolemia was found in the light LDL fractions [71]. LDL(−) was increased in the plasma of patients with high coronary heart disease risk [72]. Another study described a bimodal distribution, with LDL(−) present in both dense and light LDL fractions [73]. It has been shown, however, that the increase in the LDL(−) production was closely related to the increase in the oxidized LDL and sdLDL levels [74].

Efforts were made to detect naturally occurring modified LDL forms in human plasma. Elevated levels of Lp(a) could be selectively detected by immunoassays developed and optimized for that purpose [75]. Although oxidized LDL could not be readily isolated, other types of modified LDL have been purified, such as desialylated LDL and LDL(−). The former could be analyzed in human serum using a lectin-sorbent assay [76] and the latter by methods sensitive to the electric charge of the particles, such as ion-exchange chromatography [77] and capillary isotachophoresis [78]. The sialic acid content of isolated LDL(−) particles was 1.7-fold and 3-fold lower in healthy subjects and atherosclerosis patients, respectively, as compared to native LDL [79]. On the other hand, desialylated LDL was enriched in LDL(−) [80]. These observations suggest that desialylated and electronegative LDL subfractions might be similar or even identical (Table 2). Moreover, both desialylated and LDL(−) particles are susceptible to oxidation and contain less antioxidant vitamins than native LDL. It is therefore plausible that LDL undergoes multiple modifications in the bloodstream, starting with desialylation and acquisition of the negative charge followed by oxidation and formation of highly atherogenic and proinflammatory complexes.

## 5. sdLDL and Atherosclerotic CVD Risk

The increased atherogenicity of sdLDL is linked to the specific biochemical and biophysical properties of these particles. The small size of the particles favours their penetration into the arterial wall where they serve as a source of cholesterol and lipid storage. Longer circulation time increases the probability of atherogenic modifications of sdLDL in the blood plasma. The specific role of sdLDL, the pathogenesis of atherosclerosis, and other diseases was the subject of numerous studies [17, 81].

It has been well documented that the predominance of sdLDL (phenotype pattern B) and elevated sdLDL-C are associated with CVD risk [8, 12, 44, 82, 83]. A recent study demonstrated that sdLDL-C concentrations were a better marker for assessment of coronary heart disease (CHD) than total LDL-C [84]. In another study, elevated sdLDL-C concentrations, but not total sdLDL particle concentrations, were found to be a significant marker of CHD risk in nondiabetic individuals. In this study, sdLDL particle fraction was measured by NMR and sdLDL-C was analyzed using an automated assay in a large number of patients [85]. A smaller prospective study conducted on type 2 diabetic and prediabetic patients demonstrated that sdLDL proportion (measured by GGE) was predictive of the increase of intima media thickness (IMT) and insulin resistance [86]. Increase sdLDL level together with CA-IMT are associated with traditional risk factors for CVD. Shen et al. suggest that SdLDL-C is a better lipid variable than other standard parameters in assessing the risk of CVD using CA-IMT, even after adjustment for traditional CVD risk factors such as higher age, male sex, smoking, and family history of CVD [87]. Finally, the association of sdLDL-C with CHD has been clearly demonstrated in a large prospective study conducted on 11,419 individuals using the homogeneous assay for sdLDL assessment [56]. sdLDL-C predicted the CHD risk even in patients considered to be at low cardiovascular risk based on their LDL-C values, therefore providing an additional value for the assessment of CVD risk.

The association of sdLDL with peripheral artery disease has also been studied recently. Elevated sdLDL contents were registered in patients with worse early outcome (improved walking distance and without restenosis) after balloon angioplasty [88].

Elevated levels of sdLDL were reported in many conditions linked to atherosclerosis, such as dyslipidemia, diabetes, and metabolic syndrome (MetS), as well as in a number of other disorders [89–92]. In MetS, the increased sdLDL levels had an independent predictive value for future cardiovascular events [93]. Noteworthy, the sdLDL-C/LDL-C ratio correlated better with various parameters associated with MetS and was suggested to be a more useful clinical indicator than absolute sdLDL-C and LDL-C levels [94]. Interestingly, sdLDL fraction was significantly increased in chronic kidney disease (CKD), and its measurement could be used for CVD risk assessment in patients with CKD [95].

## 6. Effects of Statins and Other Therapies on sdLDL

As the accumulating evidence points to the important role of sdLDL in the development of atherosclerosis and CVD, many studies focus on improving the lipid profile. The predominance of sdLDL is associated with the elevated TG and decreased HDL levels [96]. Hence, the goals of the corrective therapy include lowering the proportion of sdLDL-C and/or raising the HDL-C content. Statins are widely used in clinical practice as lipid lowering agents for treatment of dyslipidemia in atherosclerosis and related disorders. Despite the large amount of information available to date, it is not yet clear whether statins are efficient for specific lowering of sdLDL-C. The results of clinical studies are sometimes contradictory in that regard [57, 82, 97]. In some studies, statins failed to decrease the sdLDL proportion because larger LDL fractions were also decreased and the ratio of sdLDL-C versus lbLDL-C was unchanged [90]. Therefore the outcome of the statin treatment should be evaluated by the absolute changes of sdLDL concentrations and not their relative content or size distributions. Lack of standardization in LDL fractionation methods and varying clinical characteristics hinder the objective comparison of the results of clinical studies. More intervention studies are necessary to draw the conclusion on the effect of statin therapy on sdLDL-C proportion and its relationship to CVD risk reduction [3].

Apart from statins, other hypolipidemic agents, such as ezetimibe and fibrates, had a beneficial effect of LDL subfractions [98]. Ezetimibe decreased the large and medium LDLs and, to a lesser extent, sdLDL particles [99]. Fibrates and niacin reduced sdLDL levels and shifted the distribution of LDL particle size towards lbLDL. Gemfibrozil lowered sdLDL fraction especially in subjects with the phenotype pattern B [100]. Fenofibrate improved the TG and HDL-C levels more efficiently than statins, and a combination therapy of fenofibrate and statins improved the lipid profile more potently than either of the medications in monotherapy. Although the pilot studies on type 2 diabetes patients failed to prove the efficacy of fenofibrate for the reduction of CHD risk, they demonstrated its beneficial effects on a number of vascular outcomes, such as retinopathy [101]. In patients with obesity, sdLDL levels can be corrected by antiobesity medications, such as orlistat and caloric restriction and changes in lifestyle [102, 103].

## 7. Conclusion

The results of recent studies demonstrate that LDL fractions have different atherogenicity, with sdLDL being more atherogenic than larger LDL subfractions. sdLDL is characterized by the enhanced ability to penetrate the arterial wall that makes it a potent source of cholesterol for the development of atherosclerotic plaque. Importantly, longer circulation times of sdLDL result in multiple atherogenic modifications of sdLDL particles in plasma, further increasing its atherogenicity. Study of the sdLDL role in the development of atherosclerosis and CVD is hindered by significant variations in LDL fractionation results obtained by different methods. The development of cheap, fast, and reliable method of quantitative LDL subfraction analysis in much needed, and significant progress has been done in that direction after the development of homogeneous assays. Statins and other lipid-lowering drugs were reported to have beneficial effects on LDL profile correction, but more studies are necessary to draw clear guidelines for sdLDL lowering in CVD prevention and treatment. Although many questions regarding the efficacy of sdLDL reduction in CVD risk management remain open, there is accumulating evidence that sdLDL-C proportion is a significant marker for CVD prediction in many conditions associated with dyslipidemia.

## Figures and Tables

**Table 1:** LDL subclasses separated by different laboratory methods.

			Small dense LDL	
Method	LDL I Large	LDL II Intermediate	LDL III Small	LDL IV Very small
UC density gradient (density)				
(a) [44]	(a) 1.019–1.023 g/ml	(a) 1.023–1.034 g/ml	(a) 1.034–1.044 g/ml	(a) 1.044–1.060
(b) [26]	(b) 1.025–1.034 g/ml	(b) 1.034–1.044 g/ml	(b) 1.044–1.060 g/ml	
UC iodixanol gradient (density)				
(a) [28]	(a) 1.016–1.028 g/ml		(a) 1.028–1.043 g/ml	
(b) [29]	(b) 1.022–1.028 g/ml		(b) 1.028–1.041 g/ml	
GGE (peak diameter)				
(a) [30]	(a) 26.35–28.5 nm	(a) 25.75–26.34 nm	(a) 22.0–25.74 nm	
(b) [45]	(b) 26.0–28.5 nm	(b) 25.5–26.4 nm	(b) 24.7–25.5 nm (III A) 24.2–24.6 nm (III B)	(b) 23.3–24.1 nm (IV A) 22.0–23.2 nm (IV B)
Ion mobility (peak size) [39]	21.9–23.8 nm	21.1–21.9 nm	20.17–21.1 nm	18.0–20.17 nm
NMR (peak size)				
(a) [34, 35]	(a) 21.3–22.7 nm	(a) 19.8–21.2 nm	(a) 18.3–19.7 nm	
(b) [32]	(b) 20.6–22.0 nm	(b) 20.4–20.5 nm	(b) 19.0–20.3 nm	
Homogenous assay [42, 43]			Separated particles with density 1.044–1.063 g/ml	
	Phenotype pattern A		Phenotype pattern B	

**Table 2:** Comparison of characteristics of sdLDL, LDL(−), and desialylated LDL.

	sdLDL	LDL(−)	Desialylated LDL
Size and density	Small dense	Small	Small dense
Aggregation/self-association	Increased^∗^ ability to self-associate		
Negative charge	Increased negative charge		
Chemical composition	Decreased sialic acid contents		
	Decreased cholesteryl esters		
	Decreased phospholipids		
	Increased protein/lipid ratio		
Amino group modification	?	Amino group modification present	
Oxidation	Increased oxidizability, reduced amount of antioxidants in the particles		
Atherogenicity	Increased atherogenicity		

^∗^In comparison to native (nonmodified) LDL.
